# Editorial: Machine Learning and Deep Learning for Physiological Signal Analysis

**DOI:** 10.3389/fphys.2022.887070

**Published:** 2022-04-13

**Authors:** Rajesh Kumar Tripathy, Mario Arrieta Paternina, José Antonio de la O Serna

**Affiliations:** ^1^ Birla Institute of Technology and Science (BITS) Pilani, Hyderabad, India; ^2^ Department of Electrical Engineering, National Autonomous University of Mexico, México City, Mexico; ^3^ Department of Electrical Engineering, Autonomous University of Nuevo León, San Nicolás de los Garza, Mexico

**Keywords:** machine learning, deep learning, physiological signals, accuracy, sensitivity

The machine learning (ML) and deep learning (DL) methods play an essential role in developing automated diagnostic systems for the accurate detection of various diseases using physiological signals (Yin et al., 2017). These methods are also used for emotion recognition, cognitive workload assessment, brain-machine interface, and other applications using physiological signals (Varshney et al., 2021; Maheshwari et al., 2021; Radhakrishnan et al., 2021; Tripathy et al., 2018). The advances of wearable technology and the internet of things (IoT) enable real-time monitoring of patients’ health status using physiological sensor data (Zhou et al., 2020). The ML and DL models are deployed on these intelligent healthcare systems for automated and real-time patient monitoring. The goal of this special issue on *Machine Learning and Deep Learning of Physiological Signal Analysis* was to disseminate the articles related to the (a) application of ML and DL for cardiovascular signal processing, (b) application of ML and DL for neural signal processing, (c) the use of ML and DL for affective computing, (d) application of ML and DL to bioinformatics, (e) ML and DL applications to human gait analysis and fatigue monitoring. In this special issue, ten papers have been submitted, and each of these papers was reviewed by at least two reviewers. Based on the reviewer’s feedback, seven papers are published in this special issue of the Frontiers in Physiology journal.

All published papers in this special issue are summarized as follows. Davis et al. have used four data preprocessing steps: quantile filtering, baseline diurnal statistics, normalization, and extract summary statistics of the physiological data to capture the features and used random forest classifier for the classification of pre-fever model versus post-fever model. They have obtained the probability of detection for three validation conditions: initial proof of concept, extension to different pathogens, and feasibility for wearable devices as 0.80, 0.90, and 0.89, respectively. Similarly, Thiam et al. have used several multi-modal supervised and self-supervised deep learning (DL) approaches to assess the pain intensity by automatically generating and measuring the physiological data. The self-supervised DL model significantly improved data efficiency and fine-tuned architecture. In another study, Fuadah and Lim have performed discrete wavelet transform (DWT) to decompose the ECG signals into several modes and extracted Hjorth descriptor features and entropy-based features. They have used K-nearest neighbor (KNN), support vector machine (SVM), random forest (RF), artificial neural network (ANN), and radial basis function network (RBFN) classification techniques to obtain the accuracy values of 100, 100, 100, 100, and 97% respectively.

Moreover, Viswakumar et al. have used the OpenPose, a 2D real-time multi-person keypoint detection technique. The system learns the associated body parts with individuals in the image using a convolutional neural network (CNN) to measure the kinematics of ankle, hip joints, and knee under dark and light conditions and subjects with long robe clothing. This system performed better than the earlier markerless systems like MS Kinect. Similarly, Adao Martins et al. performed a systematic review on fatigue monitoring through wearables using Scopus and PubMed databases and searched “fatigue,” “drowsiness,” “vigilance” or “alertness” terms in the title and proposed wearable device based systems for non-invasive fatigue quantification. Among the proposed fatigue quantification approaches, supervised machine learning models and binary classification models are predominant. Jiang et al. proposed the automatic recognition of emotion-based EEG signals during reminiscence therapy (RT) for older people. The experimental dataset of EEG signals consists of eleven older people and seven young people. The RT has been conducted by showing old public photographs to older and young people. The emotions change gradually, and a delayed effect has characterized the emotional response in EEG signals. The complex emotional features of the EEG signals are extracted using the LSTM and Bi-LSTM depth models and obtained the accuracy rate of 90.8 and 95.8%, respectively. Wang et al. explored the study of the accuracy of the different transfer learning models using the flow-volume curves to identify the ventilatory patterns. VGG13 successfully identified the ventilatory pattern with 92% of accuracy.

Finally, All three guest editors are thankful to the authors and reviewers for their contribution to the special issue *Machine Learning and Deep Learning for Physiological Signal Analysis*. We hope this research topic will further disseminate high-quality research articles in the Frontiers in Physiology journal.

## References

[B1] Maheshwari D. Ghosh S. K. Tripathy R. K. Sharma M. Acharya U. R. (2021). Automated Accurate Emotion Recognition System Using Rhythm-specific Deep Convolutional Neural Network Technique with Multi-Channel Eeg Signals. Comput. Biol. Med. 134, 104428. 10.1016/j.compbiomed.2021.104428 33984749

[B2] Radhakrishnan T. Karhade J. Ghosh S. K. Muduli P. R. Tripathy R. K. Acharya U. R. (2021). Afcnnet: Automated Detection of Af Using Chirplet Transform and Deep Convolutional Bidirectional Long Short Term Memory Network with Ecg Signals. Comput. Biol. Med. 137, 104783. 10.1016/j.compbiomed.2021.104783 34481184

[B3] Tripathy R. K. Zamora-Mendez A. De la O Serna J. A. Paternina M. R. A. Arrieta J. G. Naik G. R. (2018). Detection of Life Threatening Ventricular Arrhythmia Using Digital taylor Fourier Transform. Front. Physiol. 9, 722. 10.3389/fphys.2018.00722 29951004 PMC6008495

[B4] Varshney A. Ghosh S. K. Padhy S. Tripathy R. K. Acharya U. R. (2021). Automated Classification of Mental Arithmetic Tasks Using Recurrent Neural Network and Entropy Features Obtained from Multi-Channel Eeg Signals. Electronics 10, 1079. 10.3390/electronics10091079

[B5] Yin Z. Zhao M. Wang Y. Yang J. Zhang J. (2017). Recognition of Emotions Using Multimodal Physiological Signals and an Ensemble Deep Learning Model. Computer Methods Programs Biomed. 140, 93–110. 10.1016/j.cmpb.2016.12.005 28254094

[B6] Zhou X. Liang W. Wang K. I.-K. Wang H. Yang L. T. Jin Q. (2020). Deep-learning-enhanced Human Activity Recognition for Internet of Healthcare Things. IEEE Internet Things J. 7, 6429–6438. 10.1109/jiot.2020.2985082

